# Toll-Like Receptor Signalling Pathways and the Pathogenesis of Retinal Diseases

**DOI:** 10.3389/fopht.2022.850394

**Published:** 2022-03-31

**Authors:** Owuraku Titi-Lartey, Imran Mohammed, Winfried M. Amoaku

**Affiliations:** Academic Ophthalmology, School of Medicine, University of Nottingham, Nottingham, United Kingdom

**Keywords:** toll-like receptors, retinal diseases, age-related macular degeneration, diabetic retinopathy, ischaemic retinopathy, retinal dystrophies, inflammation, genetic polymorphisms

## Abstract

There is growing evidence that the pathogenesis of retinal diseases such as diabetic retinopathy (DR) and age-related macular degeneration (AMD) have a significant chronic inflammatory component. A vital part of the inflammatory cascade is through the activation of pattern recognition receptors (PRR) such as toll-like receptors (TLR). Here, we reviewed the past and current literature to ascertain the cumulative knowledge regarding the effect of TLRs on the development and progression of retinal diseases. There is burgeoning research demonstrating the relationship between TLRs and risk of developing retinal diseases, utilising a range of relevant disease models and a few large clinical investigations. The literature confirms that TLRs are involved in the development and progression of retinal diseases such as DR, AMD, and ischaemic retinopathy. Genetic polymorphisms in TLRs appear to contribute to the risk of developing AMD and DR. However, there are some inconsistencies in the published reports which require further elucidation. The evidence regarding TLR associations in retinal dystrophies including retinitis pigmentosa is limited. Based on the current evidence relating to the role of TLRs, combining anti-VEGF therapies with TLR inhibition may provide a longer-lasting treatment in some retinal vascular diseases.

## 1 Introduction

The retina is an important neuro-sensory organ that transduces photons of light into electrical signals and conveys visual information about our environment to the brain. Normal functioning of the retina is essential for good vision and quality of life. Retinal diseases such as diabetic retinopathy (DR), including diabetic macular oedema (DMO) and proliferative diabetic retinopathy (PDR), and age-related macular degeneration (AMD), are the leading causes of irreversible blindness worldwide (1–4). These diseases have common pathogenetic themes of inflammation, and ischaemia (5–7).

The retina is the most metabolically active tissue in the body. It is susceptible to ischaemia in situations where there is a mismatch between retinal blood flow/perfusion and retinal metabolic requirements (8), and leads to the activation of harmful compensatory mechanisms. Aberrant activation of specific innate immune responses has been implicated in the progression of retinal diseases (9), with inherited variations in genes related to immune function significantly contributing to the increased risk of disease development. The pathways and mechanisms by which immune activation and inflammation contribute to retinal diseases are not fully characterised. Microbial infections usually initiate acute inflammatory responses; however, in many retinal conditions, chronic inflammatory damage occurs due to sterile inflammation. Specifically, activation of the innate immune system is triggered when damage-associated molecular patterns (DAMPs) are released by distressed cells, which bind to pattern recognition receptors (PRR) such as toll-like receptors (TLRs) (6, 10–13).

**Figure 1:**
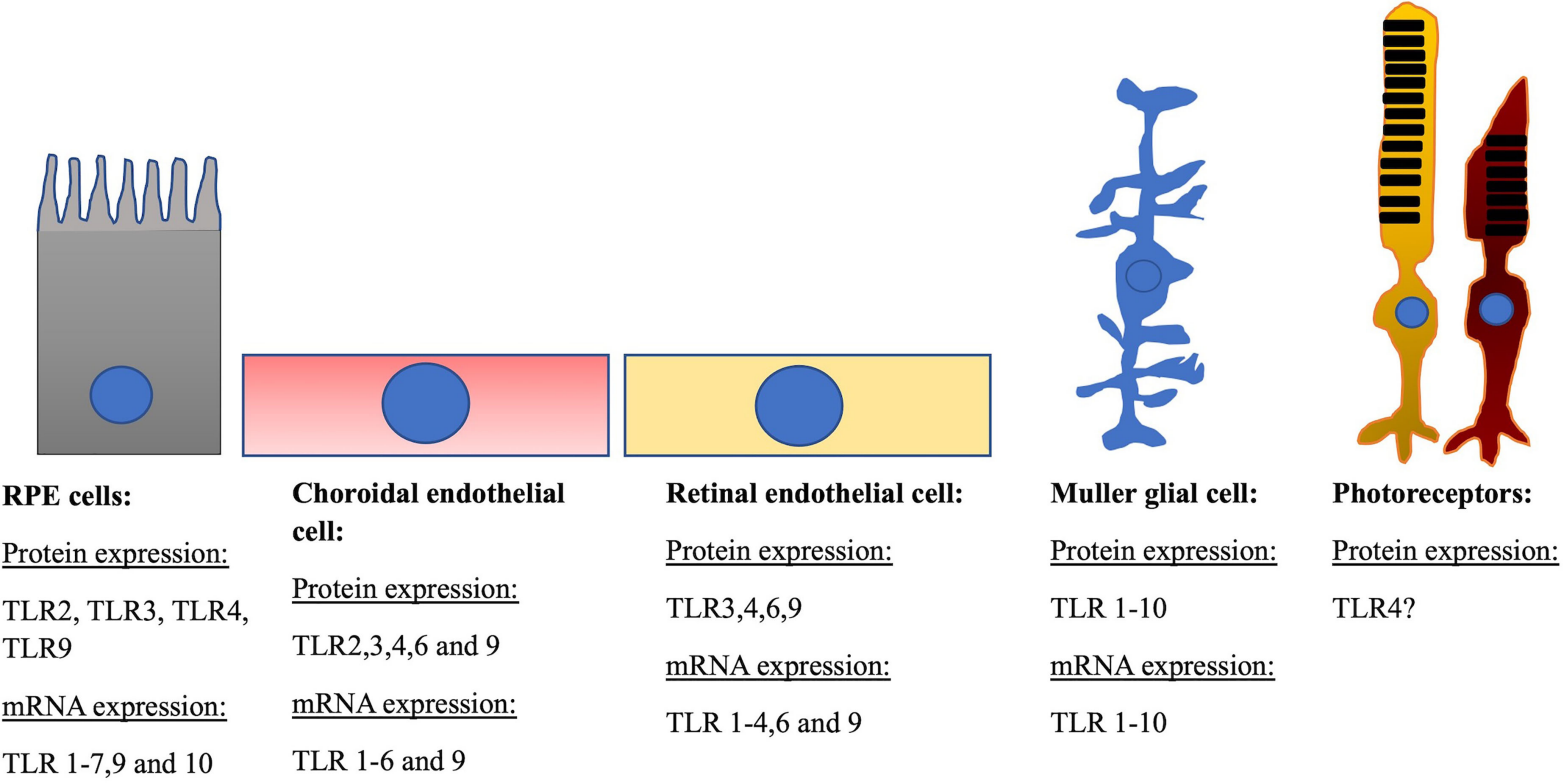
Location of TLRs on retinal cells. RPE cells: Human RPE cells express mRNA for TLRs 1-10 excluding TLR8. TLRs 1 and 3 being the most highly expressed at mRNA level. Protein of TLR 2-4, and -9 have also been identified. Choroidal endothelial cell: Human choroidal endothelial cells express mRNA for TLRs 1-6 and 9 and protein for TLRs 2-6 and 9. Retinal endothelial cell: Human retinal endothelial cells express mRNA for TLRs 1-4, -6 and -9 and protein for TLRs 3, 4, 6 and 9 (17). Muller glial cell: Human muller cells express mRNA and protein for TLRs 1-10. Photoreceptors: Photoreceptors from the 661W murine cell line express TLR4 protein.

TLRs are expressed in several ocular tissues (14–16), and cells including the retinal pigment epithelium, and vascular endothelial cells of the retina and choroid (**Figure 1**) (17–19). Knowledge on TLR expression and pathways in the choroido-retinal complex have continued to expand. In addition, there has been a significantly improved understanding of the pathophysiology of different retinal diseases, including contributions from chronic inflammation, and hypoxia-activated signalling pathways. This review aims to provide a detailed landscape of TLRs regulation/interaction in the causation of retinal diseases.

### 1.1 Toll-Like Receptors

Nüsslein-Volhard and Wieschaus first discovered the Toll gene in 1980 as a mutated gene that led to changes during the embryonic development of the drosophila fly (20). Later, studies into the mechanisms used by the Drosophila fly to fight against and recognize infections contributed to the discovery of Toll protein and subsequently, TLRs function in the innate immunity of other species (21, 22).

**Figure 2:**
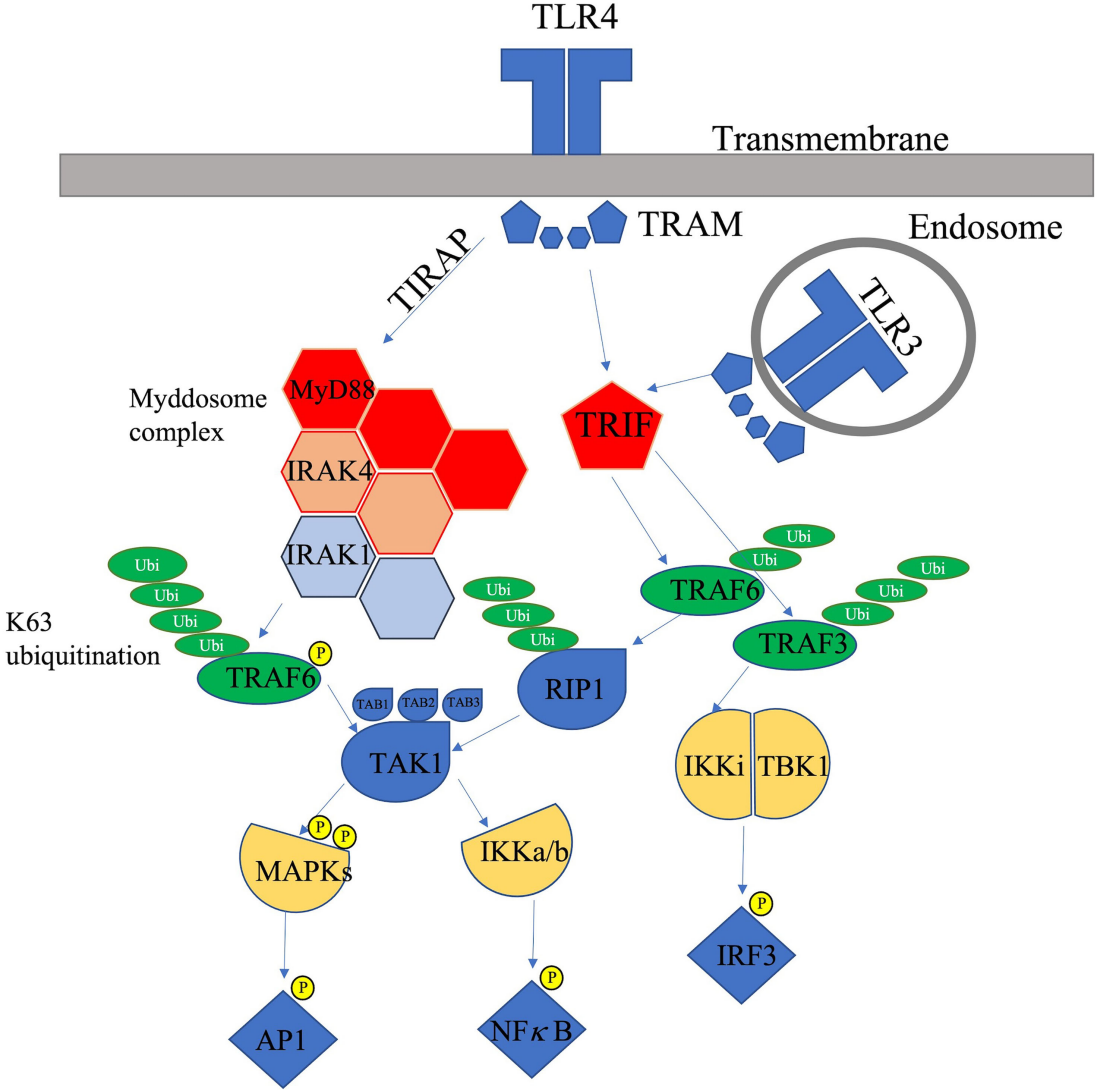
TLR-3 and TLR-4 signalling pathways [adapted from (34)]. TLR3 receptors are located intracellularly in endosomal compartments whereas TLR4 is located on the cell surface. Activation of the TLRs initiate signalling pathways downstream of two adaptor proteins, TRIF and MyD88. Activation of the TIRAP pathway leads to formation of the myddosome complex which comprises MyD88, IRAK 4 and IRAK1. Activated IRAK1 induces TRAF6 after k63 polyubiquitination, this leads to activation of TAK1 which subsequently activates the IKK complex-NF-κB and MAPK. Activation of the TRAM pathway *via* the TRIF adaptor leads to the activation of TRAF6 and TRAF3, TRAF6 recruits the RIP1 kinase which activates the TAK1 complex which subsequently triggers the MAPK and NF-κB signalling pathways. The IKK related kinases, IKKI and TBK1, are recruited by TRAF3 and this leads to phosphorylation of IRF3 leading to Type 1 interferon transcription.

TLRs are a family of type-1 transmembrane receptors (23) that contribute to the activation of innate and adaptive immunity through inflammatory mediators produced in response to molecular patterns associated with micro-organisms (**Table 1** and **Figure 2**) known as pathogen-associated molecular patterns (PAMPs). In addition, TLRs can also respond to self-derived molecules, also known as damage-associated molecular patterns (DAMPs), released from cells as a result of aging or injury. DAMPs include self-derived molecules such as HMGB-4, nucleic acids, uric acid crystals, hyaluronan, surfactant protein A and other extracellular matrix products (35).

**Table 1:** Ligand, activation pathway and location of human toll like receptors.

	Ligand	Activation pathway	Cellular location	Ref
TLR -1	Bacterial lipoproteins, lipoteichoic acids	MyD88	Cell surface	Jin et al. (24), Ling and Xiong (25)
TLR -2	Lipoproteins/Lipopeptides (Various pathogen) Peptidoglycan/Lipoteichoic acid (Gram-positive bacteria) Lipoarabinomannan (Mycobacteria) Glycosylphosphatidylinositol (Trypanosoma cruzi) Zymosan	MyD88	Cell surface	Jin et al. (24) Chang et al. (26)
TLR -3	Viral dsRNA Self RNA Polyinosine:polycytidylic acid [poly(I:C)]	TRIF	Endosomal	Lim et al. (27)
TLR - 4	Lipopolysaccharides	MyD88/TRIF	Cell surface	Poltorak et al. (28), Chang et al. (26)
TLR – 5	Flagellin	MyD88	Cell surface	Gewirtz et al. (29)
TLR – 6	Bacterial lipoproteins	MyD88	Cell surface	Mulfaul et al. (9)
TLR – 7	Single stranded RNA	MyD88	Endosomal	Lund et al. (30)
TLR – 8	Single stranded RNA	MyD88	Endosomal	Gorden et al. (31)
TLR – 9	Bacterial and viral DNA rich in unmethylated CpG-DNA motifs	MyD88	Endosomal	Kezic and McMenamin (32)
TLR - 10	diacylated lipopeptides	MyD88	Cell surface	Fore et al. (33)

The TLR family consists of 10 members in humans (TLR1-10) (**Table 1**) and 12 members in mice (TLR1-LTR9, TLR11-TLR13) (36). The location and distribution of TLRs vary in different cell types. TLR1-2, TLR4-6 and TLR10 are found predominantly on the extracellular surface, whereas TLR3, TLR7-9, TLR11-13 (which are only found in mice) are found intracellularly (**Figure 2**).

TLRs consist of a horseshoe-shaped ectodomain containing leucine-rich repeats (LRRs), which function to recognize PAMPs, a transdomain component and an intracellular cytoplasmic Toll/IL1 (TIR) domain that triggers signalling of downstream molecules (34). The ectodomain portion of TLRs form either a homo or heterodimer. Upon binding of a PAMP or DAMP to the ectodomain of a TLR, specific TIR domain-containing adaptor proteins such as TIR domain-containing adapter inducing interferon-β (TRIF) or Myeloid differentiation factor 88 (MyD88) are recruited. These trigger the initiation of signalling pathways that lead to the activation of molecules such as nuclear factor kappa-B (NF-κB), type-1 interferons, or IRFs (34). The signalling pathways mediated by TLRs is divided into two groups: TRIF dependent or MyD88 dependent.

TLR2 forms a heterodimer with TLR1 leading to recognition of tri-acyl lipopeptides (26), whereas the heterodimer formed between TLR2 and TLR6 recognizes di-acyl lipopeptides (26). TLR10, the most recently discovered, is the only TLR with anti-inflammatory properties (33), and can detect the influenza A virus (37) and works with TLR2 to detect *Listeria* spp. (38).

#### 1.1.1 MyD88 Pathway

Activation of MyD88 associated TLRs leads to activation of Toll/Interleukin-1 receptor domain-containing adapter protein (TIRAP), which conducts signal from the TLR2 and 4 to MyD88 (**Figure 2**). Recruitment of MyD88 after ligand binding to corresponding TLRs leads to MyD88 forming a ‘myddosome’ complex with interleukin-1 receptor-associated kinase (IRAK) family members (39). MyD88 recruits IRAK4, leading to the formation of the MyD88-IRAK4 complex which subsequently recruits the substrates of IRAK4, IRA2 or IRAK1. This leads to the formation of a myddosome complex bringing the IRAKs kinase domains into proximity for phosphorylation and activation (39).

IRAK4/1 recruits tumour necrosis factor (TNF)-receptor-associated factor (TRAF)6, which along with ubiquitin-conjugating enzyme 13 (UBC13), leads to the k63 ubiquitination of TRAF6 (40). The ubiquitination of TRAF6 leads to the recruitment of TAK-1 binding proteins (TAB1,2,3), which form a TAK-1 protein kinase complex that activates the IκB kinase (IKK)–NF-κB complex and MAPK kinases through phosphorylation (41). The binding of TAK1 to the IKK complex leads to activation of the IKK complex and subsequent activation of NF-κB mediated genes (42).

#### 1.1.2 TRIF Pathway

The binding of ligands to TRIF-associated TLRs leads to the interaction of the receptor adaptor, TRIF, with TRAF-3 and -6 (43). TRAF-6 subsequently recruits the RIP-1 kinase, which activates the TAK1 complex, ultimately triggering the activation of NF-κB and MAPK pathways (44) and induces the production of inflammatory cytokines. TRAF3 activates and recruits the IKK*i*, TBK1, and NEMO, which phosphorylates IRF3 and induces the expression of type 1 interferon (IFN) associated genes.

#### 1.1.3 TLRs Localization in Human Retina

TLRs are widely expressed on a variety of immune cells such as dendritic cells, natural killer cells, and B-cells. They are also found on non-immune cells such as fibroblasts, endothelial and epithelial cells (34).

In humans, RPE cells have been shown to express genes for TLRs 1-7, -9 and TLR10 (18, 26, 45) with TLR1 and -3 being shown to be highly expressed. Proteins of TLR2 (18, 46), TLR3 (18, 45), TLR4 (18, 47, 48) and TLR9 (45) have also been localized in human RPE cells.

Our group has previously demonstrated the mRNA expression of TLRs 1-6 and 9 in primary human choroidal endothelial cells (hCEC) (17). Utilizing western blotting, we quantitated the protein expression of TLRs -3, -4 and -9 in hCEC, whilst with flow cytometry, TLR -3, -4, -6 and -9 were shown to be localized on the surface of cells and intracellularly (17). Subsequently, Feng and co-workers confirmed the localization of TLR2 in hCECs (46). In primary human retinal endothelial cells (hREC), mRNA for TLRs 1-4, 6 and 9 were expressed (17). In addition, TLRs -3, -4, -6, and -9 were shown to be localized both on the surface and intracellularly in hREC (17). Expression of key TLRs were further validated in hCECs and hRECs using synthetic PAMPs. Overall, this study showed for the first time that TLRs are localized in retinal and choroidal endothelial cells and potentially may be involved in the inflammatory damage of retina during DR and AMD. Human Muller cells have been shown to express mRNA and protein for TLR 1-10, MyD88, TRIF, TRAM and TRAF6 along with MD2 and CD14 co-receptors (49). Photoreceptors from the 661W murine cell line have been shown to express TLR4 protein (50).

### 1.2 Literature Search: Characteristics of Selected Studies

We performed a literature search of the National Library of Medicine (PubMed) and Ovid MEDLINE(R) from July 1946 to August 2021 using the following search criteria. Only full-text articles published in English were included. ((retinal or inflammatory retinal or inflammatory retinopathy or diabetic retinopathy or hypertensive retinopathy or age-related macular degeneration, AMD or ARMD or birdshot chorioretinopathy or Vogt-Koyanagi-Harada or VKH or retinal vascular occlusion or central retinal vein occlusion or central retinal artery occlusion or branch retinal vein occlusion or branch retinal artery occlusion or retinitis pigmentosa or cystoid macular oedema) and (toll-like receptor or TLR or toll-like)).

**Figure 3:**
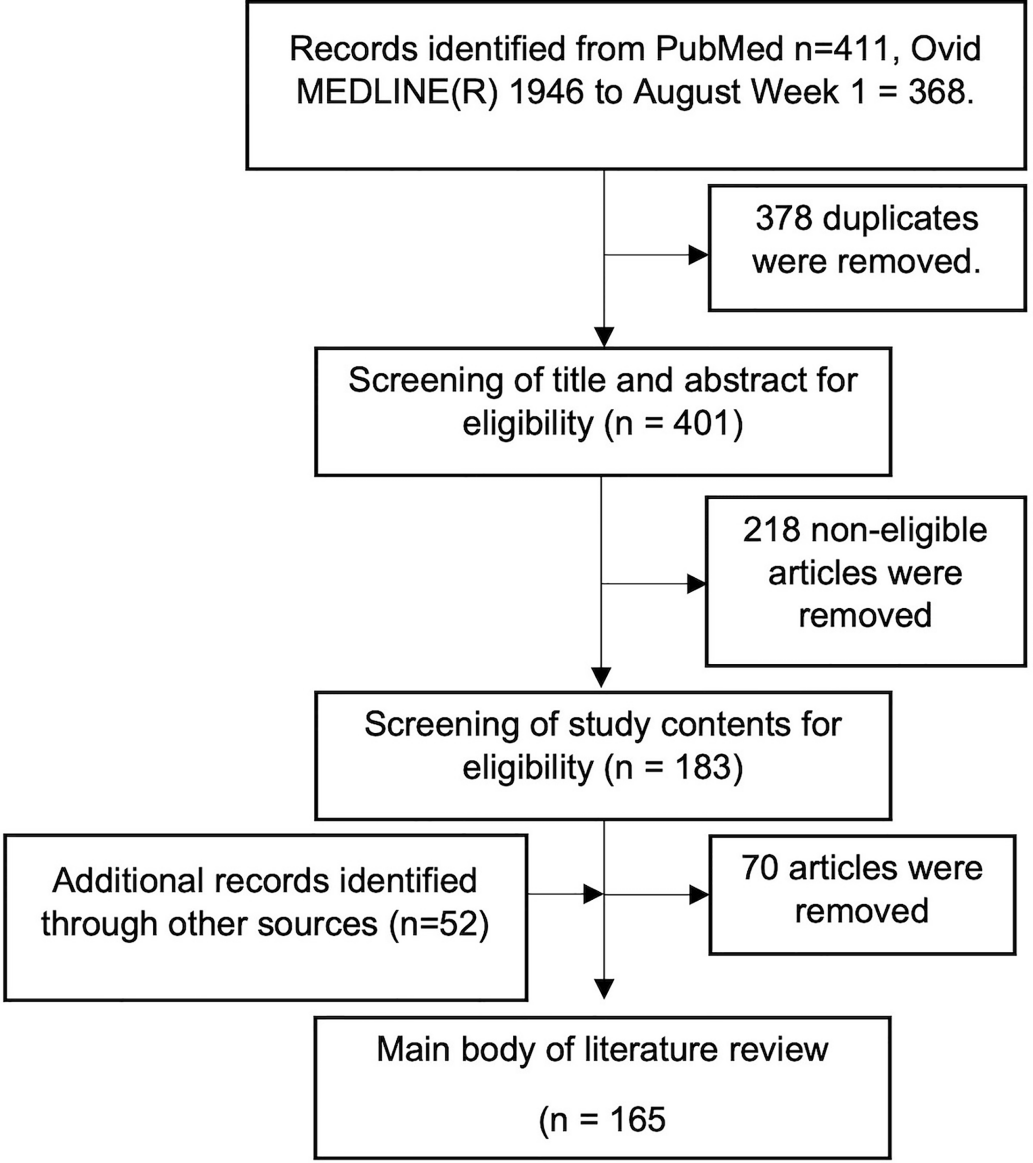
Flow chart showing the article selection process.

Our initial search criteria yielded 779 studies (**Figure 3**). After removing duplicates, we obtained 401 studies. Screening of the title and abstract yielded 183 studies as unrelated studies, and editorial/commentaries were removed. Further screening by review of article contents yielded 113 studies as the main body of our literature review, between January 2004 and September 2021. Another 52 articles were acquired by direct database search.

## 2 Retinal Diseases and TLRs

### 2.1 Diabetic Retinopathy

#### 2.1.1 Background

Diabetes mellitus (DM) can be divided into two broad categories: type 1 and type 2 diabetes. Type 1 DM occurs due to a complex interaction between genetic and environmental factors, resulting in autoimmune destruction of the beta cells in the islet of Langerhans. Type 2 DM also occurs due to a complex interaction between genetic and environmental factors, resulting in reduced insulin sensitivity (51) and eventual beta-cell depletion in the later stages of type 2 DM. Both types subsequently lead to a loss of homeostatic control of blood glucose levels.

DM is one of the modern world’s most significant epidemics, affecting both developing and developed countries, estimated to affect approximately 464 million adults worldwide (20-79 years of age) in 2019, with a projection to increase to 700 million by 2045 (52). Historically, DM was perceived as a disease that mainly affected developing countries. However, 79% of adult patients with DM are from low or middle-income countries (53).

DM is associated with various vascular complications varying from microvascular diseases affecting the retinal vasculature, renal vasculature, or microvasculature of the cerebral circulation. Vascular pathology in blood vessels supplying nerves can contribute to the development of diabetic neuropathy. It can also have deleterious effects on larger vessels, contributing to peripheral limb ischaemia, coronary artery disease, and carotid artery stenosis.

Diabetes causes a wide range of ocular diseases, the most severe and commonest (54) being DR, which is also a leading cause of blindness in the developed world (55, 56). The risk of developing DR is related to the duration of DM (57) but also influenced by diabetic control (58), high blood pressure (59), and high blood lipid levels (60).

#### 2.1.2 Pathophysiology of Diabetic Retinopathy

The blood-retinal barrier (BRB) prevents the passage of harmful molecules from the systemic circulation into retinal tissue (61). The blood-retinal barrier consists of an inner and outer BRB. The inner BRB is formed by tight junctions between the continuous endothelium lining retinal vessels, and the outer BRB is formed by the tight junctions between RPE cells (61).

The RPE consists of a single layer of cuboidal epithelial cells that lie between the photoreceptors and choroid (47), they aid with removal of waste products and secretes cytokines/chemokines to facilitate cellular messaging. Aged or damaged photoreceptor outer segments are constantly shed and digested by RPE cells (47).

Various pathological mechanisms compromised the BRB in diabetes. Increased paracellular permeability due to disruption of tight junction proteins such as occludin (61), which is frequently reported as being associated with diabetes-induced BRB failure (62). Endothelial cell loss occurs due to leucostasis, oxidative stress, and endothelial progenitor dysfunction (61).

Pericyte loss contributes to the progression of diabetic retinopathy due to a loss of the regulation of blood flow through the contractile properties of pericytes and loss of endothelial cell maintenance (61). Loss of retinal endothelial cells can lead to vascular occlusions and create a hypoxic cellular environment, which subsequently enhances the secretion of angiogenic factors and the growth of abnormal retinal vessels (proliferative DR). Rupture of these abnormal vessels, lead to vitreous hemorrhage or result in tractional retinal detachment. Proliferative/late-stage DR is frequently managed with laser photocoagulation and anti-VEGF monoclonal antibody therapy (63).

Activation of TLR signalling pathways has been shown to contribute to the development of diabetes-related renal complications. TLR4/NFκB pathway mediated activation of peroxisome proliferator-activated receptor-y coactivator-1a (PGC-1a) has been shown to accelerate the development of diabetic kidney disease (64).

DR is associated with a chronic subclinical inflammatory state, characterised by elevated levels of inflammatory proteins such as heat shock protein (HSP) 60/70, fibrinogen, oxidized LDL, and fibronectin (55). It is thought that activation of PRR involved in the innate immune response, such as receptor for advanced glycation end-product (RAGE), may be associated with the development of diabetes or the manifestation of diabetic complications.

#### 2.1.3 Toll-Like Receptors in Diabetic Retinopathy

##### 2.1.3.1 Leucocyte Adhesion

Diabetes is associated with increased expression of TLR4 (63, 65–72), TLR2 (65, 68, 73) and TLR7 (74) in human and mouse retinal cells. Hyperglycaemia has been shown to increase MyD88 independent and dependent signalling (75) along with pro-inflammatory/angiogenic mediators downstream to MyD88 such as NFκB, VEGF, TNF-α and IL-1β (65, 67–69, 72, 75).

Deleting MyD88 or TLR2/4 from bone marrow-derived immune cells significantly reduces diabetes-induced pathological changes in the retina such as leucostasis, vascular permeability, superoxide generation and intercellular adhesion molecule 1 (ICAM-1) (76). Bone marrow-derived leukocytes from mice with diabetes have been shown to kill endothelial cells more than those from naïve mice (76). A subsequent further study confirmed these phenotypic differences of BM cells. TLR4 deficient BM cells from diabetic mice showed reduced features of DR compared with diabetic wild-type mice (77). TLR4 deficient mice showed lower levels of inflammatory cytokines and angiogenic factors. Notably, transplantation of TLR4 sufficient BM-derived cells from wild type mice into TLR4 deficient mice leads to restoration of pro-inflammatory cytokine (TNF-α, IL-1β) release and angiogenesis-related genes (VEGF, HIF-1α) (77).

**Figure 4:**
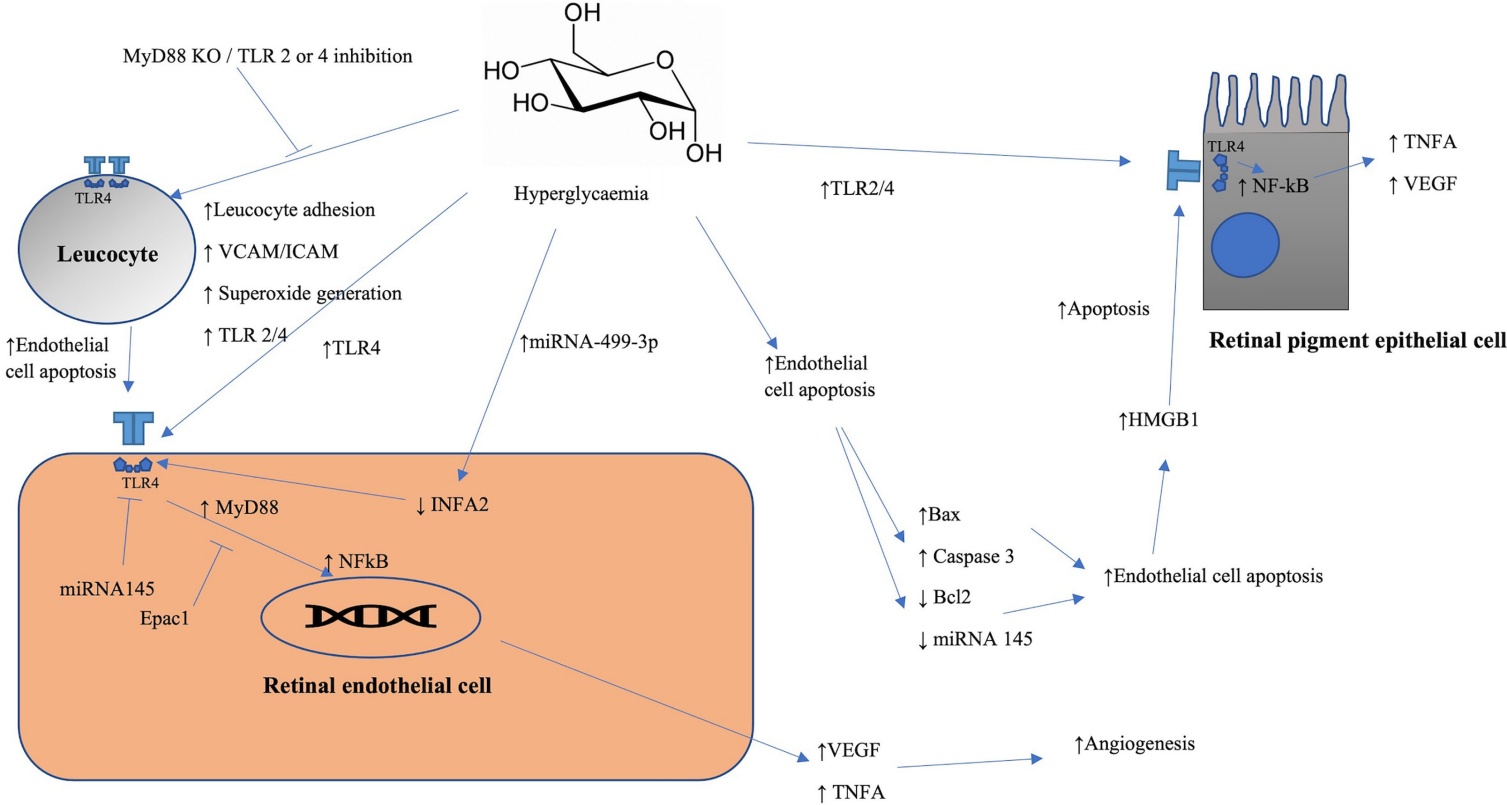
Schematic representation of TLRs involvement in diabetic retinopathy. Initiation of the pathological endothelial changes seen in diabetes are dependent on TLR-2 and -4. HMGB1 levels are raised in response to hyperglycaemia in rat endothelial cells and the source may be from bone marrow derived cells or from dying endothelial cells. HMGB1 binds to the TLR4 receptor initiating activation of the NF-κB pathway leading to subsequent increased levels of TNF-α and VEGF in RPE cells. Diabetes induced miRNA-499-3p triggers the levels of TLR4 *via* inhibition of INFA2 leading to reduced endothelial cell proliferation and increased apoptosis. Retinal endothelial cell apoptosis is associated with raised levels of markers of apoptosis such as bax and caspase-3 and reduction of anti-apoptotic markers such as BCL-2. Hyperglycaemic condition exacerbates the levels of TNF-α, NF-κB and VEGF production in ARPE19.

There is a substantial amount of evidence showing increased expression of TLRs, in particular TLR4, in models of diabetes in both human and mouse retinal cells, along with increased expression of molecules downstream to TLR. Overall, hyperglycaemic conditions appear to increase the activity of leucocytes in the retinal vascular endothelium in a TLR4-dependent fashion (**Figure 4**). To summarize, hyperglycaemic conditions appear to increase the activity of leucocytes in the retinal vascular endothelium in a TLR-4 dependent fashion; however, other TLRs are seen to be increased in both mouse and human retinal cells.

##### 2.1.3.2 Endothelial Cell Apoptosis

High mobility group box -1 (HMGB1) is a nuclear protein present in the nucleus of most cells and is secreted by macrophages, natural killer cells and mature dendritic cells in response to cellular distress or injury (78). Levels are raised in response to hyperglycaemia in rat retinal endothelial cells and ARPE-19 cells (65). HMGB1 has been shown to decrease insulin receptor and Akt phosphorylation *via* signalling through TLR4 in human retinal endothelial cells (79).

Hyperglycaemia is also associated with hREC apoptosis (80, 81), reduced RPE viability (82) and raised levels of TLR4 and NF-κB (81) in HRECs. The effect of which is inhibited by the application of gastrodin *via* induced activation of the SIRT1/TLR4 pathway (81). Hyperglycaemia has been shown to increase TNF-α, NF-κB activity and VEGF production in ARPE19 cells (65). This is alleviated by the administration of antibodies against HMGB1 (65). Hyperglycaemia-induced increased levels of TLR4 and HMGB1 are inhibited by the application of the Box A portion of HMGB1 (Box A) and glycyrrhizin (83) in both cultured human REC and mouse retina.

Hyperglycaemia induced endothelial cell apoptosis is associated with raised levels of apoptotic markers such as Bax and cleaved Caspase-3, whilst anti-apoptotic markers such as BCL-2 are reduced (80). Application of Box A and glycyrrhizin to REC was associated with reduced insulin receptor substrate (IRS-1)^Ser307^ phosphorylation and reduced cleavage of caspase 3 (83). Microglial matrix metalloproteinase 9 (MMP-9) levels are raised in response to hyperglycaemia *via* HMGB1/TLR4 signalling (84) in BV2 murine glial cells.

TLR4 signalling in RECs is attenuated by exchange protein activated by cAMP 1 (Epac1) leading to inhibition of hyperglycaemia induced MyD88-mediated signalling, suggesting that Epac1 acts upstream of TLR4 dependent signalling (75). In addition, the loss of Epac1 has been shown to increase the levels of inflammatory mediators downstream to TLR4 (NFκB, TNF-α, IL-1β) (75).

Diabetic rat retinal cell have decreased IFNα2 expression associated with upregulated micro-RNA-499-3p (71). The miRNA-499-3p reduces IFNα2 levels by targeting 3’-UTR of IFNα2, which subsequently reduces the retinal cell proliferation and lead to increased apoptosis.

Inhibition of miRNA-27a has been shown to increase the caspase-3/9 activity and induces expression of IL-6, TNF-α, and IL-1β in human RPE-1 cells exposed to high glucose levels, which are associated with reduced cell viability (82). miRNA-27a transfection of RPE cells lead to TLR4 inhibition, reduced cell viability and levels of IL6, TNF-α, IL-1β, respectively (82).

TLR4 deletion in diabetic mice reduces VEGF and glial fibrillary acidic protein (GFAP) levels and retinal tissue thickness compared to the naïve mice (70, 77). This is further associated with the reduced retinal inflammation which is characterised by the reduced levels of IL6, TNF-α, and IL-1β (70). Hyperglycaemia induced apoptosis and oxidative stress in hRECs is reduced by the overexpression of miRNA-145 (80) which targets TLR4 (80).

TLR7 deficiency attenuates hyperglycaemia induced retinal damage in mice RPE cells (74) and has an anti-angiogenic effect associated with reduced vascular tufts, fluorescein leakage and retinal hemorrhages (74). In addition, TLR7 deficiency in DR mouse model also reduces the pro-inflammatory cytokines such as TNF-α, IL-1β, IL-6, IL-12 in retina (74).

Excess calorie consumption and/or obesity contributes to the risk of developing type-2 diabetes and metabolic syndrome (85). Administration of a high-fat diet to TLR4 knockout and wild type mice revealed that the high-fat diet induces increased oxidative stress and DNA damage in the inner retina in a TLR4-dependent fashion (85).

Activation of β-adrenergic receptor (β-AR) by administering an agonist compound-49b (67) inhibits diabetes-activated TLR4 signalling in mice retina. In addition, compound-49b also reduces TLR4-signalling activation in human retinal endothelial, and Muller cells cultured in high glucose conditions (67).

Diabetic rats show higher levels of insulin like growth factor (IGF), VEGF, TNF-α, glutamate, and malondialdehyde (MDA), from retinal supernatants, which were shown to be reduced by the administration of metformin (63). The expression of mRNA for NFκB, TLR4, and TNF-α are elevated in diabetic rat retinas and implicated with the reduced thickness of the cell layers and ganglion cell vacuolization, both of which are reduced by the administration of metformin (63). The authors implicated that the effect of metformin is mediated *via* the attenuation of TLR4-induced oxidative stress.

TLR4 has been shown to regulate permeability in murine retinal endothelial cells (86, 87) and the loss of which reduces diabetes-induced thinning of murine retinal vasculature (86, 87) and retinal vascular damage, respectively (86).

Hyperglycaemia has been shown to be associated with increased production of DAMPs such as HMGB1. This subsequently leads to activation of TLR mediated apoptotic pathways and raised levels of markers of apoptosis such as Bax and caspase 3. Hyperglycaemia is also associated with raised levels of pro-inflammatory cytokines *via* a TLR dependent mechanism. This subsequently leads to loss of retinal endothelial cells, a crucial step in the pathophysiology of diabetic retinopathy.

##### 2.1.3.3 Endothelial Cell Permeability

Diabetes has been shown to induce retinal endothelial permeability in Cdh5Cre-Epac1^f/f^ mice, the effect of which is reduced up on administration of a TLR4 antagonist, TAK-242 (75). TLR4 has been shown to regulate the levels of occludin and ZO-1 (tight-junction proteins) in murine retinal endothelial cells (86).

Increased endothelial cell permeability allows large plasma proteins such as low-density lipoproteins (LDLs) to cross the blood-retinal barrier (BRB), which can subsequently get oxidized in the retina (72). The retina is especially susceptible to oxidative damage due to its high energy demand, high amounts of polyunsaturated fatty acids and exposure to UV radiation (63). This can lead to the production of reactive oxygen species (ROS) (88), which can generate oxidized lipids through lipid peroxidation.

Oxidized LDLs can trigger inflammation in murine retinal Muller cells by stimulating the release of pro-inflammatory cytokines such as IL-6, TNF-α, and IL-1β (72, 88). Oxidized LDL also triggers the production of DAMPs which can activate TLRs leading to retinal inflammation. Raised levels of oxidized LDL are associated with reduced Muller cell survival (72), which is reversed by inhibition of a TLR4 co-receptor complex protein, myeloid differentiation protein-2 (MD2) (72).

In murine Muller cells, TLR4 knockout in mice leads to increased phosphorylation of the insulin receptor (89, 90) along with decreased phosphorylation of Insulin receptor substrate 1 (IRS-1) and caspase 3 (90). Hyperglycaemia induced impairment of the insulin receptor and Akt phosphorylation in rMC-1-O (rat Muller) cells is reduced on the administration of TLR4 siRNA, implying high glucose induces dysfunctional insulin signalling *via* TLR4 in retinal Muller cells (90).

Changes in retinal endothelial permeability are a crucial part of the pathophysiology of diabetic retinopathy. TLR4 appears to play a role in regulation of endothelial permeability *via* levels of intercellular adhesion molecules such as occluding and ZO-1. This facilitates the passage of pro-inflammatory DAMPs such as oxidized lipids triggering further inflammation and cellular damage.

##### 2.1.3.4 Platelet-Rich Plasma

Rat platelets exposed to high glucose levels are associated with increased levels of CD61 and Annexin-V positive platelet-rich plasma exosomes (PRP-exos) (91). PRP-exos are taken up by retinal endothelial cells and, diabetic PRP-exos increases the production of MDA and ROS whilst reducing the activity of superoxide dismutase (SOD) (91). Increased levels of ICAM-1 and VCAM-1 when compared to PRP-exos from non-diabetic rats (91) were also reported, indicating endothelial injury *via* the activation of TLR4.

PRP-exos were associated with increased levels of TLR4, MyD88, and NF-κB/p65, which were inhibited by a TLR4 receptor antagonist (TAK-242) (91). This also reverses the PRP-exo induced MDA, ROS, ICAM1, and VCAM1 levels, respectively. Moreover, TAK-242 also reduces PRP-exos triggered blood vessel leakage (91). PRP-exo stimulation of TLR4 was shown to be mediated *via* CXCL10 (91).

#### 2.1.4 Toll-Like Receptor Genetic Polymorphisms in Diabetic Retinopathy

DM is a multifactorial condition and various factors contribute to the risk of development and progression of DM and its complications, including inherited risk factors. Although numerous laboratory-based studies demonstrated that the effect of TLRs activation in DR is quite complex, the genetic association studies demonstrating the relationship between TLRs and DR risk are far less and scarce. In humans, the TLR4-Asp299Gly polymorphism has specifically shown an association with the DR risk (**Table 2**).

**Table 2:** Reports of association between TLR4 polymorphism and diabetic retinopathy.

Research group	Year	Country	Ethnicity	Receptor	Genotyping method	Polymorphism (Reference SNP Cluster Report)	Sample size		Case			Control		
							Case	Control	Ho 1 (%)	Het (%)	Ho2 (%)	Ho 1 (%)	Het (%)	Ho 2 (%)
Buraczynska et al. (55)	2009	Poland	Caucasian	TLR4	PCR restriction fragment length polymorphism	rs4986790 (Asp299Gly)	352	140	309 (88) AA	40 (11) AG	3 (1) GG	130 (93) AA	10 (7) AG	0 (0) GG
Singh et al. (51)	2014	India	North Indian	TLR4	PCR restriction fragment length polymorphism	rs10759931	128	320	56 (43.7) AA	72 (56.3) AG	0 (0) GG	173 (54.1) AA	144 (45.0) AG	3 (0.9) GG
Singh et al. (51)	2014	India	North Indian	TLR4	PCR restriction fragment length polymorphism	rs1927914	128	320	61 (47.7) TT	66 (51.5) TC	1 (0.8) CC	184 (57.5) TT	134 (41.9) TC	2 (0.6) CC
Xu et al. (56)	2015	China	Han	TLR4	PCR-restriction fragment length polymorphism	rs10759931	139	274	55 (39.5) AA	81 (58.3) AG	3 (2.2) GG	115 (42.0) AA	151 (55.1) AG	8 (2.9) GG
Xu et al. (56)	2015	China	Han	TLR4	PCR-restriction fragment length polymorphism	rs1927911	139	274	9 (6.5) TT	67 (48.2) TC	63 (45.3) CC	22 (8.1) TT	158 (57.7) TC	94 (34.2) CC
Xu et al. (56)	2015	China	Han	TLR4	PCR-restriction fragment length polymorphism	rs1927914	139	274	37 (26.6) TT	83 (59.7) TC	19 (13.7) CC	108 (39.4) TT	147 (53.6) TC	19 (7.0) CC
Buraczynska et al. (92)	2016	Poland	Caucasian	TLR4	automated cycle sequencing in CEQ 8000 Genetic Analysis System	rs4986790 (Asp299Gly)	342	748	292 (85.5) AA	48 (14) AG	2 (0.5) GG	695 (93) AA	50 (6.6) AG	3 (0.4) GG
Zaharieva et al. (93)	2017	Bulgaria	Bulgarian	TLR4	PCR-restriction fragment length polymorphism	rs4986790 (Asp299Gly)	10	75	7 (70) AA	3 (30) AG	0 (0) GG	71 (94.7) AA	4 (5.3) AG	0 (0) GG
Aioanei et al. (94)	2019	Romania	Caucasian	TLR4	polymerase chain reaction-restriction fragment length polymorphism	rs4986790 (Asp299Gly)	198	200						
Aioanei et al. (94)	2019	Romania	Caucasian	TLR4	polymerase chain reaction-restriction fragment length polymorphism	rs4986791 (Thr399Ile)	198	200						

##### 2.1.4.1 Asp299Gly Polymorphism

A cohort of 864 patients with T2DM, 352 had DR, was compared to 420 healthy individuals. The carriage of the GG and AG genotypes (299Gly) in the rs4986790 polymorphism was found to be associated with a higher rate of diabetic retinopathy (p<0.001) as carriers of this genotype (63%) had a higher rate of DR compared to non-carriers (39%). The 299Gly genotype of the rs4986790 polymorphism was associated with a higher risk of early-onset DR compared to late-onset DR (OR = 5.0, 95% CI = 2.33–10.71) (55).

Another cohort of 1090 patients with T2DM, of which 342 had DR, and 748 did not have DR, was compared to 716 healthy individuals. There was a significant association between the Asp299Gly G allele and the risk of developing diabetic retinopathy in diabetic patients (p = 0.0002, OR = 2.12, 95% CI = 1.43-3.12) (92).

Another study included a cohort of 113 patients with type 2 diabetes, 10 of which had DR, were compared to 75 patients with T2DM without DR. There was a significant association between the Asp299Gly polymorphism and DR (p=0.018, OR =7.61, 95% CI = 1.41–41.08) (93).

A cohort of 198 patients with T2DM, including 120 with DR, was compared to 200 healthy controls. The Asp299Gly polymorphism appeared to have a protective effect against diabetic retinopathy, contrary to previous studies (94).

The Asp299Gly TLR4 polymorphism has been reported to show an increased TNF-a response to LPS stimulation (95). Furthermore, crystallography has revealed that TLR4 has two highly preserved regions involved in ligand binding (95). The Asp299Gly polymorphism is located close to the TLR4-MD2 binding area, it does not appear to have a direct effect on the binding location but is thought to increase the peptide bonds rotation freedom and a loss of negative charge at position 299 (95).

##### 2.1.4.2 Others

A cohort of 128 patients with DR were compared to 320 healthy controls. The TLR4 polymorphism rs10759931 (p = 0.05, OR = 1.50, 95% CI 0.99–2.26) and rs1927914 (p = 0.05, OR = 1.48, 95% CI 1.0–2.24) were significantly associated with the development of DR when compared to the control group (51). No haplotypes were associated with either susceptibility or resistance to DR (51).

A cohort of 198 patients with T2DM, of which 120 had DR, was compared to 200 healthy controls. The Thr399Ile (P<0.001, OR=0.487, 95% CI=0.211-0.648) polymorphisms appeared to provide a protective status against DR when compared to the healthy controls (94).

### 2.2 Age-Related Macular Degeneration

#### 2.2.1 Background Information

Age-related macular degeneration (AMD) is the commonest cause of vision loss in patients above the age of 50 (25, 48, 96), affecting almost 200 million people worldwide (1), which is projected to increase due to an ageing population in the developed world. AMD is usually divided into the dry form, the end stage of which is geographic atrophy (GA) or wet form (neovascular AMD) (3, 97, 98).

Although the exact etiology of AMD is not clear, the most significant risk factor is age with smoking being the biggest modifiable risk factor (88) amongst many others, such as hypertension (48), family history and body mass index.

Several genes are associated with AMD risk. The age-related maculopathy susceptibility 2 (*ARMS2*) and high-temperature requirement A serine peptidase (*HTRA1*) genes are found on the 10q26 (Ch10) locus (99), which has the greatest contribution to the genetic risk of developing AMD. Other genes such as complement factor H (*CFH*) and complement factor H-related genes (*CFHR*) 1 to 5 which are located on the 1q32 (Ch1) region (99), are associated with a risk of developing AMD. Polymorphisms in the TLRs have also been shown to be related to the risk of AMD development (100).

It is clinically divided into two categories; early (visual symptoms are not significant) and late (usually associated with significant vision loss). AMD can also be divided according to the pathological features (of neovascularisation and vascular leakage) as wet (exudative/neovascularization associated) or dry (non-exudative), with the majority of patients suffering from dry (90%) (101) compared to wet (10%) AMD.

The initial identifying feature of dry AMD is the occurrence of soft drusen; these are extracellular deposits located below the RPE consisting of protein- and lipid-rich cellular debris (5). The accumulation of extracellular materials results in the development of low-grade chronic inflammation (102) because of the activation of innate immune system. The natural history of dry AMD manifests as geographic atrophy of the RPE (25). It occurs due to confluent regions of photoreceptor and RPE cell death (103). In more than half of patients, it is bilateral i.e., affecting both eyes. The average rate at which geographic atrophy propagates is between 1.3 to 2.8 mm^2^ per year (96).

Neovascular or wet AMD is one of the leading causes of blindness in the elderly population in developed countries, affecting over 1 million patients in the United States (98). It is characterised by the development of abnormal choroidal neovascularisation (CNV) or retinal angiomatous proliferation (RAP), which invade the retina leading to fluid leakage (25, 104).

#### 2.2.2 Toll-Like Receptors in AMD

##### 2.2.2.1 Cell Viability

Stimulation of TLR3 and TLR4 using their respective ligands, poly I:C and lipopolysaccharide (LPS) on porcine RPE cells significantly decreases RPE cell viability and the levels of IL-6 and IL-8 (105). Poly I:C inhibits wound healing of a damaged RPE after prolonged treatment, whereas LPS enhance wound healing (105). TLR3 activation by poly I:C reduces porcine RPE cell viability, which is mediated *via* JNK activation (106).

Intra-vitreous administration of siRNA targeting Cdh16 (kidney-specific cadherin 16), and bone-specific osteocalcin (Bglap1) into wild type mice inhibits the choroidal neovascularization (CNV) (107, 108). siRNAs that target Vegfr1 (AGN211745) and VEGFA (bevasiranib) have been shown to inhibit CNV formation. Interestingly, this effect is lost in TLR-3 deficient mice (107) implying that specific siRNA inhibits CNV *via* cell surface TLR-3. The use of siRNA molecules for the treatment of AMD appears to be very appealing; however, intravitreal administration of siRNA molecules, regardless of target or sequence, have been shown to induce retinal degeneration characterised by disruption of the retinal pigmented epithelium. This is dependent on TLR-3 activation leading to caspase-3 mediated cell death (109), thereby limiting the potential use of siRNA molecules in the treatment of CNV.

Stimulation of TLR3 using poly I:C, on mouse photoreceptor cells causes significant structural damage and functional loss characterised by reduced a-wave and b-wave amplitudes on the electroretinograph (110).

CD36 is an obligate co-receptor for TLR2 (111). CD36 deficiency in Cx3cr1 deficient background mice reduces the age- or photo-stress induced accumulation of subretinal mononuclear phagocytes and subretinal inflammation (111).

ATP Binding Cassette Subfamily A Member 4 (Abca4)/Retinol Dehydrogenase 8 (Rdh8) deficient mice were shown to develop AMD-like phenotypes on prolonged photosensitisation (112). When compared to wild-type mice, light exposure in Abca4/Rdh8 deficient mice led to increased expression of TLR2 and 4 in association with elevated pro-inflammatory/chemotactic cytokines such as *CCL2, CR2, CCCL12, Cx3cr1, IL-1b*, *TNF-α*, *CFH*, and *VEGFA* genes (112). Deletion of TLR4 resulted in milder retinal degeneration in this model (112), which concluded that endogenous ligand release from photoreceptor injury/death triggers retinal inflammation *via* TLR4 signalling activation.

TLR2/4 deficiency has been associated with increased area of subretinal fibrosis compared to wild type mice (113). Similar phenotype was also observed with the application of anti-TLR2/4 antibodies (113). Notably, administration of recombinant HSP70 increased intraocular IL-10 levels and significantly reduced the subretinal fibrosis in TLR2/4 deficient mice (113). These studies have implicated the pathologic role of TLR-2, -4, and -3 signalling pathways in the progression of AMD disease phenotype in murine models.

The pathophysiology of AMD includes the accumulation of extracellular debris and subsequent cellular apoptosis. This cellular debris may contain DAMPs which triggers the immune responses *via* TLRs leading to reduced RPE and photoreceptor viability.

##### 2.2.2.2 Oxidative Stress

Oxidative stress is a key step in the pathogenesis of AMD, due to the high metabolic requirements of the retina and direct exposure to light. With age, RPE cells lose their inherent abilities to digest photoreceptor outer segments which result in lipofuscin accumulation leading to oxidative stress (9, 46).

Inhibition of TLR2 limits photo-oxidative stress-induced retinal degeneration and outer nuclear layer thinning (88) in murine retina, and is associated with reduced complement C3 deposition in photoreceptors (88). Photo-oxidative stress was shown to activate TLR2 *via* endogenous DAMP production, which induces the production of C3 and further activation/deposition (88).

Previous studies showed that patients with AMD have 4.6 times higher levels of CEP-Eps (114). Exposure of human primary RPE cells *in vitro* to oxidized lipids, carboxyethyl pyrrole (CEP) and Pam2CSK4 (PAM) in combination increased *IL6, MCP1, CXCL8 and TLR2* gene expression (46) compared to Pam2CSK4 alone. However, CEP alone treatment did not affect these genes (46). Furthermore, TLR2 blockage suppressed CNV initiation and progression in their mouse model and that blockage of TLR2 and VEGF resulted in additive effects on CNV suppression.

Endogenous lipid peroxidation may lead to production of DAMPs. The 7-ketocholesterol (7KCh) is formed by auto-oxidation of cholesterol and is associated with Alzheimer’s disease, atherosclerosis, and AMD, respectively (115). Treatment of ARPE19 cells with 7KCh induces pro-inflammatory molecules such as IL-1β, CAAT-enhancer-binding protein homologous protein (CHOP), IL-8 and VEGF, which is attenuated by administration of a TLR4 inhibitor, CLI-095. In addition, CLI-095 also inhibits 7KCh induced angiogenesis *in vivo* (115).

Sodium iodate (NaIO_3_), a potent oxidizing agent, was shown to activate the complement alternative pathway causing degradation of photoreceptors (88), RPE fragmentation, deposition of complement C3 fragments and induction of *TLR4* mRNA in wild-type mice (116). In addition, T*LR2* deficient mice following NaIO_3_ exposure reduced the RPE degeneration and preserved ONL layers (88). Overall, this suggests that TLR2 plays an important role in oxidative damage in the retina in response to NaIO_3_.

Activation of the TLR-3 receptor by poly I:C during paraquat-induced oxidative stress modifies murine photoreceptor (117) and murine RPE (118) cell viability in a STAT3-dependent manner (117, 118).

In a co-culture of murine photoreceptor cells and Muller cells exposed to H_2_0_2_ induced oxidative stress, the activation of TLR4 receptor with LPS has been shown to further increase the photoreceptor loss more than when compared to H_2_0_2_ alone treatment (119). On the other hand, the Wnt3a treatment showed protective effect against H_2_0_2_ induced photoreceptor loss. If Wnt3a is incubated along with LPS in an H_2_0_2_ induced oxidative stress model, the protective effect of Wnt3a appears to be lost. TLR4 activation is thought to regulate the Wnt3a *via* decreased phosphorylation of the Wnt receptor LRP6 (119).

With age, the ability to process extracellular debris is lost, accumulation of extracellular debris can contribute to increased photo-oxidative stress mediated *via* TLR dependent pathways.

##### 2.2.2.3 Inflammatory Pathways

ARPE-19 cells exposed to poly I:C was shown to increase the activation of TLR3 signalling and RelA production. Notably, repeated exposure with poly I:C increases RelA levels above that induced by the initial exposure (120). It is thought that the initial poly I:C exposure primes ARPE-19 cells to produce higher amounts of RelA. Activation of TLR3 regulates RelA levels *via* TBK1/IKKϵ and JAK/STAT pathways (120). Poly I:C treatment also leads to increased expression of IL6, IL8, TNF-α, MCP1, ICAM-1 and VEGF in a dose-dependent manner (121). Activation of TLR3 on porcine RPE cells is also associated with a dose-dependent cellular apoptosis and VEGF release (106).

An amyloid protein, Aβ-42, has been detected in AMD drusen (122). Treatment of ARPE19 cells with Aβ-42 peptides increases IL-6, IL-8, IL-33 and VEGF expression (122) *via* the activation of TLR4/MyD88/NFκB signalling cascade, the effect of which is reduced by TLR4 inhibition (122).

Nuclear factor erythroid 2-related factor 2 and peroxisome proliferator-activated receptor-gamma coactivator 1-alpha (NFE2L2/PGC1α) are usually involved in the regulation of antioxidant production. Deficiency of this complex was shown to increase the accumulation of drusen-like extracellular material in sub-RPE region (102) with a significant increase noted in TLR3/TLR9 levels in mice retina (102). This was also associated with the increased levels of complement component C5a (102).

##### 2.2.2.4 Choroidal Neovascularization

The laser-induced CNV area in mice retina is enlarged by the application of TLR2 agonists, Pam2CSK4 (123, 124) and *Chlamydia pneumoniae* antigen or zymosan (123). Administration of anti-VEGF antibodies reduced laser-induced CNV area whilst CCL2 deletion increases the lesion area (124). *Chlamydia pneumoniae* antigen has also been shown to induce IL6 and VEGF *via* activation of TLR4/MyD88 axis (123).

TLR2 is involved in the recruitment of murine leucocytes to CNV (124) and increased levels of pro-inflammatory cytokines (46). In addition, increased levels of TLR3 are also detected on RPE cells within human CNV membranes (125). Systemic administration of the TLR-2 agonist Pam2CSK4 (PAM) in CNV mouse model leads to enlargement of CNV size compared to mice treated with saline control (124). Inhibition of TLR2 signalling by intravitreal injection of anti-TLR2 antibodies in murine eyes has been shown to suppress CNV formation, this suppressive effect is enhanced when anti-VEGFR2 is co-administered with anti-TLR2 antibodies (46).

The expression of TLR2/3 mRNA and protein in serum mononuclear leucocytes cells of patients with neovascular AMD is higher compared to the healthy controls. When the serum mononuclear leucocytes from patients with neovascular AMD are exposed to the TLR-2 ligand, PGN, they produce higher levels of IL6 and IL8. In addition, IL6 is also elevated in leucocytes from nAMD patients when exposed to the TLR-3 ligand poly IC compared to leucocytes from healthy controls (126).

Stromal cell-derived factor (SDF-1), also known as CXCL12, participates in CNV *via* CXCR4 and CXCR7 (127). LPS induces CXCR4 and CXCR7 levels in a choroid-retinal endothelial cell line (Rf/6A), derived from rhesus monkeys, *via* the activation of TLR4 (127). The transcription of CXCR4 and CXCR7 was regulated by the phosphorylation of ERK 1/2 and NFκB (127).

CNV is a defining feature of wet age-related macular degeneration. It has been shown to be influenced by activation of TLR-2 signalling pathway.

#### 2.2.3 Toll-Like Receptor Genetic Polymorphisms in AMD

There are several studies into the association of TLR genetic polymorphisms with AMD (**Table 3**). There is a degree of inconsistency in the literature, with different studies reporting different results. This may be accounted for by the variation in ethnic origin of the patients involved in the studies.

**Table 3:** Reports of association between TLRs polymorphism and retinal degeneration.

Research group	Year	Country	Ethnicity	Receptor	Genotyping method	Polymorphism (Reference SNP Cluster Report)	Sample size		Case			Control		
							Case	Control	Ho 1 (%)	Het (%)	Ho2 (%)	Ho 1 (%)	Het (%)	Ho 2 (%)
Zareparsi et al. (48)	2005	United States (Michigan)	Caucasian	TLR4	Allele specific PCR and restriction digestion	D299G (rs4986790)	667	439	584 (87.76) (DD)	81 (12.1) (DG)	2 (0.3) GG	412 (94.1)	25 (5.7) (DG)	1 (0.2) (GG)
Zareparsi et al. (48)	2005	United States (Michigan)	Caucasian	TLR4	Allele specific PCR and restriction digestion	T399I (rs4986791)	667	439	513 (85.4) TT	86 (14.3) TI	2 (0.3) II	221 (93.2) TT	16 (6.8) TI	0 (0) II
Kaur et al. (128)	2006	India	N/a	TLR4	Allele specific PCR and restriction digestion	D299G (rs4986790)	120	120	n/a	n/a	n/a	n/a	n/a	n/a
Kaur et al. (128)	2006	India	N/a	TLR4	Allele specific PCR and restriction digestion	T399I (rs4986791)	120	120	n/a	n/a	n/a	n/a	n/a	n/a
Despriet et al. (129)	2008	Netherlands	Caucasian	TLR4	Denaturing high performance liquid chromatography (DHPLC)	D299G (Rs4986790)	368	368	(90.3) AA	(9.4) AG	(0.3) GG	(89.3) AA	(10.7) AG	() GG
Despriet et al. (129)	2008	Netherlands	Caucasian	TLR4	Denaturing high performance liquid chromatography (DHPLC)	T399I (rs4986791)	368	368	(89.7) CC	(10.0) CT	(0.3) TT	(87.7) CC	(12.3) CT	() TT
Despriet et al. (129)	2008	Netherlands	Caucasian	TLR4	Denaturing high performance liquid chromatography (DHPLC)	K354K	368	368	(98.0) AA	(2.00) AG	(0.00) GG	(87.7) CC	(12.3) CT	(0.00) TT
Despriet et al. (129)	2008	United states	Caucasian	TLR4	TaqMan assay	D299G (rs4986790)	357	173	(88.5) AA	(10.7) AG	(0.8) GG	(90.7) AA	(9.0) AG	(0.3) GG
Edwards et al. (130)	2008	United States (Dallas)	Caucasian	TLR3	TaqMan assay	rs3775291 L412F	396	181	171 (44.2) CC	185 (47.8) CT	31 (8.0) TT	92 (57.9) CC	56 (35.2) CT	11 (6.9) TT
Edwards et al. (130)	2008	United States (Dallas)	Caucasian	TLR7	TaqMan assays	rs179008 Gln11Leu	396	181	151 (60.9) AA	81 (32.7) AT	16 (6.5) TT	72 (77.4) AA	17 (18.3) AT	4 (4.3) TT
Edwards et al. (130)	2008	United States (Michigan)	Caucasian	TLR3	TaqMan assay	rs3775291 L412F	611	323	280 (46.1) CC	275 (45.2) CT	53 (8.7) TT	158 (49.8) CC	124 (39.1) CT	35 (11.0) TT
Edwards et al. (130)	2008	United States (Michigan)	Caucasian	TLR7	TaqMan assay	rs179008 Gln11Leu	611	323	238 (62.1) AA	118 (30.8) AT	27 (7.1) TT	97 (58.1) AA	58 (34.7) AT	12 (7.2) TT
Yang et al. (131)	2008	United States (Utah)	Caucasian	TLR3	SNaPshot on an ABI 3100XL	rs3775291 L412F	825	359	406 (49) CC	368 (44) CT	51 (6.2) TT	156 (43.5) CC	163 (45.4) CT	40 (11.14) TT
Yang et al. (131)	2008	United States	Caucasian	TLR3	SNaPshot on an ABI 3100XL	rs3775291 L412F	450	421	243 (54.0) CC	176 (39.1) CT	31 (6.9) TT	183 (43.5) CC	196 (46.6) CT	42 (10.0) TT
Cho et al. (132)	2009	United States, Australia	Caucasian	TLR3	Taqman SNP Genotyping Assay	rs3775291 L412F	687	936	331 (48.2) CC	299 (43.5) CT	57 (8.3) TT	460 (49.1) CC	380 (40.6) CT	96 (10.3) TT
Cho et al. (132)	2009	United States, Australia	Caucasian	TLR4	Taqman SNP Genotyping Assay	rs4986790	687	936	592 (87.8) CC	76 (11.3) AG	6 (0.9) GG	819 (88.2) AA	98 (10.5) AG	12 (1.3) GG
Klein et al. (96)	2010	AREDs participants	AREDs participants	TLR3	Taqman genotyping platform	rs3775291 L412F	114	448	54 (47.4) GG	46 (40.4) AG	9 (7.9) AA	n/a	n/a	n/a
Sng et al. (133)	2011	Singapore	Chinese	TLR3 vs polypoidal choroidal vasculopathy	automated bidirectional DNA sequencing	rs3775291 L412F	120	274	19 (15.8) TT	67 (55.8) TC	34 (28.3) CC	3 (14.2) TT	130 (47.4) TC	105 (38.3) TT
Sng et al. (133)	2011	Singapore	Chinese	TLR3 vs choroidal neovascularization	automated bidirectional DNA sequencing	rs3775291 L412F	126	274	19 (15.1) TT	63 (50.0) TC	44 (34.9) CC	3 (14.2) TT	130 (47.4) TC	105 (38.3) TT
Cheng et al. (134)	2014	China	Chinese	TLR3	PCR amplification and automated DNA sequencing	rs5743303	96	96	67 (69.8) AA	27 (28.1) AT	2 (2.1) TT	56 (58.3) AA	28 (29.2) AT	12 (12.5) TT
Cheng et al. (134)	2014	China	Chinese	TLR3	PCR amplification and automated DNA sequencing	rs5743305	96	96	41 (50.0) TT	32 (39.0) TA	9 (11.0) AA	56 (58.3) TT	40 (31.7) TA	0 AA
Cheng et al. (134)	2014	China	Chinese	TLR3	PCR amplification and automated DNA sequencing	rs5743312	96	96	60 (65.2) CC	25 (27.2) CT	7 (7.6) TT	56 (58.3) CC	24 (25.0) CT	16 (16.7) TT
Cheng et al. (134)	2014	China	Chinese	TLR3	PCR amplification and automated DNA sequencing	rs3775291 L412F	96	96	27 (58.7) CC	17 (37.0) CT	2 (4.3) TT	52 (54.2) CC	36 (37.5) CT	8 (0.8) TT
Cheng et al. (134)	2014	China	Chinese	TLR3	PCR amplification and automated DNA sequencing	rs3775290	96	96	44 (49.4) CC	38 (42.7) CT	7 (7.9) TT	36 (37.5) CC	60 (62.5) CT	0 TT
Cheng et al. (134)	2014	China	Chinese	TLR3	PCR amplification and automated DNA sequencing	rs6830345	96	96	3 (3.3) TT	20 (21.7) TC	69 (75.0) CC	0 TT	28 (29.2) TC	68 (70.8) CC
Sharma et al. (135)	2014	India	North India	TLR3	TaqMan SNP genotyping assay	rs3775291 L412F	115	61	6 (5.0) AA	33 (30) AG	73 (65.0) GG	0 AA	27 (44.0) AG	34 (56.0) GG
Guven et al. (136)	2015	Turkey	Caucasian	TLR2	Real time PCR	rs5743708	183	200	152 (83) GG	31 (17) GA	0 (0) AA	190 (95) GG	10 (5) GA	0 (0) AA
Guven et al. (136)	2015	Turkey	Caucasian	TLR4	Real time PCR	rs4986790	183	200	172 (94) AA	10 (5) AG	1 (1) GG	196 (98) AA	3 (1) AG	1 (1) GG
Guven et al. (136)	2015	Turkey	Caucasian	TLR4	Real time PCR	rs4986791	183	200	183 (100) CC	0 (0) CT	0 (0) TT	196 (98) CC	2 (1) CT	2 (1) TT
Ling and Xiong (25)	2019	China	Han	TLR4	Polymerase chain reaction-restricted fragment length polymorphism	rs1927914 C>T	138	146	56 (40.58) (TT)	71 (51.45) (CT)	11 (7.97) (CC)	51 (34.93) (TT)	67 (45.89) (CT)	28 (19.18) (CC)
Ling and Xiong (25)	2019	China	Han	TLR4	Direct sequencing method	rs1927907 C>T	138	146	67 (48.55) (CC)	60 (43.48) (CT)	11 (7.97) (TT)	80 (54.79) (CC)	51 (34.93) (CT)	15 (10.27) (TT)

##### 2.2.3.1 TLR4

TLR4 is a prominent mediator of inflammatory pathways and regulator of cholesterol efflux through its stimulation by LPS which is regulated by APOE and ATP-binding cassette transport-1 (ABCA1) (48). Furthermore, altered binding of DAMP or changes in TLR related signal transduction pathways could lead to a loss of the immune privilege of the choroid/outer retina leading to subsequent accumulation of extracellularly deposited materials.

A cohort of 667 patients with AMD and 439 healthy controls were investigated. The frequency of the G allele in the TLR4-D299G polymorphism was higher in patients with AMD compared to the control (P = 0.001, age/sex-adjusted OR = 2.42, 95% CI = 1.43-4.08) (48). The frequency of the TLR4-T399I polymorphism was higher in patients with AMD compared to the control (P = 0.003, age/sex-adjusted OR = 2.37, 95% CI 1.33-4.22) (48). However, when both polymorphisms were included in a multiple logistic regression model, only the TLR4—D299G G allele was associated with a significant risk of AMD (P = 0.025, OR = 2.65, 95% CI = 1.13-6.25) (48).

A cohort of 120 patients with AMD was compared to 120 healthy controls. The TLR4-D299G and TLR4-T399I polymorphisms were not found to be associated with a significant risk of AMD (128).

A cohort of 368 patients with AMD and 368 healthy controls in the Netherlands were investigated, a significant association between D299G, T399I and K354K TLR4 polymorphisms and the risk of AMD was not found (129). The same article also investigated a cohort of 357 patients with AMD against 173 healthy controls from the United States, a significant association between AMD risk and the D299G, T399I and K354K TLR4 polymorphisms were not found (129).

A combined study based on three cohorts totalling 687 patients with AMD and 936 healthy controls was performed. No association was found between TLR4-*rs4986790* in the three independent cohorts individually or combined (132).

The study by Guven et al. (136) included a cohort of 183 patients with AMD and were compared to 200 healthy control subjects. A significant association was not found between the risk of AMD and the TLR4-Asp299Gly and TLR4-Thr399Ile genotypes (136).

Ling and Xiong (25) investigated TLR associations in 138 patients with AMD and 146 healthy control subjects. No significant difference between the frequency of TLR4-rs1927914 in patients with AMD compared to the healthy control subjects (25) were found. There was a significant difference in the CC genotype of TLR4-rs1927914 between AMD patients and healthy controls with the CC genotype and allele appearing to be protective AMD (P = 0.010 OR= 0.358, 95% CI=0.162–0.791), (P = 0.039, OR = 0.698, CI = 0.497-0.983) (25).

A meta-analysis of the association between TLR4 polymorphisms and AMD risk was performed. It revealed an association between the TLR4-rs4986790 polymorphism and AMD in a heterozygote and dominant model (AG vs. AA, OR = 1.400, 95%CI = 1.049–1.867, P = .022), (GG+AG vs. AA, OR = 1.365, 95%CI = 1.028–1.813, P = .032) respectively (100). No association was found between the TLR4- rs4986791 polymorphism and AMD risk (100).

##### 2.2.3.2 TLR3

A multistage study was performed involving three cohorts of patients. The first cohort, based in Dallas, USA, consisted of 396 patients suffering from AMD against 181 controls; 10 polymorphisms across TLRs 1,3-7,10 were identified, however, only the TLR3 polymorphism (rs3775291 L412F, P = 0.01) and TLR7 polymorphism (rs12663316 Xp22.3, P = 0.02) were associated with AMD (130), these were found to not be significant after correction for multiple testing was applied (130). The TLR3-L412F and TLR7- Xp22 polymorphisms were investigated in two other cohorts no significant association was found (130, 131).

TLR3 polymorphisms were studied in a cohort of 825 patients with AMD and compared to 359 healthy control subjects (131). The TLR3 polymorphisms rs5743303 and rs3775291 were investigated, and the risk of GA, CNV and soft drusen development was analysed. No significant association between TLR3- rs5743303 and AMD was found. A significant association between the ‘T’ allele of rs3775291 and a lower risk of geographic atrophy was identified (*p=0.005, additive allele-dosage model, Orhet=0.712, 95% CI 0.503 -1.00; Orhom=0.437, 95% CI 0.227-0.839* (131). These results were replicated in a cohort of 450 patients with AMD, and 421 controls, where a significant association between rs3775291 and geographic atrophy (p=0.000543) (131) was identified. A second replication on a cohort of patients from AREDS consisting of 184 patients with AMD and 134 healthy controls further confirmed the relationship between rs3775291 and GA (p=0.002). A combined analysis of the three cohorts yielded an association (p= 0.000000124, FDR adjusted) between rs3775291 and GA, which was highly significant (131).

A combined study based on three cohorts totalling 687 patients with AMD and 936 healthy controls was performed. No association was found between TLR3-rs3775291 in the three independent cohorts individually (132).

In a cohort of 114 AMD patients and 448 healthy control subjects, no significant association between the risk of AMD and the TLR4- rs3775291 polymorphism was found (genotypic OR = 1.55, CI = 0.77-1.67, genotypic P = 0.149, allelic OR = 1.22, CI 0.896-1.67, allelic P = 0.233) (96).

Similarly, a cohort of 126 patients with CNV and 120 patients with polypoidal choroidal vasculopathy (PCV) was compared to 274 healthy controls, and no significant association between the TLR3-rs3775291 polymorphism and CNV in AMD was found genotypic p = 0.698, allelic = 0.5847) (133).

Cheng et al. (134) included 96 patients with AMD and 96 healthy control subjects in a study where the TLR3 gene was sequenced in its entirety, revealing 6 polymorphisms: rs5743303, rs5743305, rs5743312, rs3775291, rs3775290, and rs6830345. No significant difference was found between the distribution of TLR3 polymorphisms in the AMD cases versus the control group (134).

Sharma et al. (135) compared TLR3 polymorphisms in a cohort of 115 patients with AMD were compared to 61 healthy controls. No significant difference was found between the distribution of TLR3-rs3775291 GG or AG genotypes and AMD (OR value = 0.093, p = 0.112, CI = 0.005–0.1.73, OR = 0.163 and p = 0.222, CI = 0.009–2.99, respectively) (135).

A meta-analysis of the association between TLR3-rs3775291 polymorphism and AMD risk showed that the T allele of the TLR3-rs3775291 polymorphism was associated with a reduced risk of GA (p = 0.04, OR = 0.78, 95% CI: 0.62-0.98) (137). TLR3-rs3775291 was also associated with neovascular AMD (p = 0.01, OR = 0.78, 95% CI: 0.64–0.94) (137), and overall associated with AMD in a recessive model (p = 0.03, OR = 0.88, 95% CI: 0.79–0.99).

The TLR3-rs3775291 polymorphism does not appear to affect the mRNA, surface expression or protein of TLR3 (103), but the binding capacity of the polymorphism is 51.12 ± 3.96% (P<0.001) (103) compared to the wild type; subsequently reduced expression of TLR3 mediated NF-kB may explain the reduced risk of developing GA with this polymorphism.

##### 2.2.3.3 Other TLRs

A cohort of 183 patients with AMD was compared to 200 healthy control subjects. The TLR2-Arg753Gln genotype was associated with a significant risk of developing AMD (OR= 3.88; 95% CI: 1.76–8.75, p = 0.001) (136).

### 2.3 Ischaemic Retinopathy

#### 2.3.1 Background Information

Ischaemia is caused by the reduced blood supply to a localized area due to the obstruction of blood vessels, which subsequently leads to cellular hypoxia and eventual cell death (138). In the retina, ischaemia leads to neuronal cell death, activation of glial cells, and release of cytotoxic intercellular mediators (138).

Retinal ischaemia occurs in various conditions such as diabetic retinopathy, retinal vein/artery occlusion, and ocular ischaemic syndrome, which can lead to subsequent pathological blood vessel formation, otherwise known as neovascularization. Neovascularization is linked to the initiation of inflammatory pathways by the production and release of proangiogenic cellular mediators by TLRs through induction of angiogenic factors such as VEGF (139). Following a period of retinal ischaemia, restoration of blood supply may be associated with reperfusion injury, due to exhaustion of intracellular energy stores and subsequent mitochondrial dysfunction causing the production of large amounts of ROS. Reperfusion of ischaemic tissue can lead to increased levels of these factors resulting in retinal damage (140).

The second most common retinal vascular disease (after diabetic retinopathy) is retinal vein occlusion, which may be either central (CRVO) or branched (BRVO) depending on the site of the occlusion.

#### 2.3.2 The Role of TLRs in Retinal Ischaemia and Neovascularization

Several animal models have been used to simulate retinal ischaemia. These include timed elevation of intraocular pressure, followed by reperfusion (138). Other models of retinal ischaemia include clipping retinal vessels for 30 minutes and then subsequently removing the clip (13).

##### 2.3.2.1 Inflammatory Pathways

Raised levels of heat shock protein 70 (Hsp70) have been detected in the vitreous humour of mice exposed to retinal ischaemia-reperfusion injury (141). Extracellular Hsp70 was associated with RGC death *in vitro*, which was reduced on the administration of a protein kinase C (PKC) inhibitor in a glial-RGC co-culture (141). This indicates that the high levels of extracellular HSP70 induce a pro-inflammatory response when PKC is simultaneously activated. This was mediated *via* the TLR4-MyD88 pathway leading to TNF release (141).

HMGB1 is a nuclear protein present in the nucleus of most cells (142). It is secreted by macrophages, natural killer cells, and mature dendritic cells in response to cellular distress or injury (79, 142). HMGB1 levels are raised following the ischaemic-reperfusion (IR) injury (142) and accumulate in the vitreous humour (143).

HMGB1 inhibition (143) in murine RGCs or gene deletion (142) reduces IR induced retinal damage whilst the direct administration of recombinant HMGB1 increases the loss of RGCs in retina (143). HMGB1 initiates inflammatory pathways mainly *via* TLR4 activation (142, 143).

Microglial cell recruitment and subsequent inflammatory cytokine release is initiated by activation of TLRs. TLR-1 to -3 are elevated (138, 139, 144) in the mouse retina and enhances TLR3-dependent microglial cells recruitment following IR injury. Besides, TLR3 activation also increases the inflammatory cytokines such as IL-6, IL-1b, TNFa, TGFB in mouse retina following IR injury (138). Ischaemia increases caspase-3 reactivity in the ganglion cell layer (GCL) of mouse retina (138), which was mainly induced *via* TLR4-mediated proinflammatory cytokines (138).

Models of oxygen-induced retinopathy (OIR) in rats have been shown to increase the levels of TLR3 (145) and NF-κB. OIR induced TLR3 expression was significantly upregulated up on the administration of poly I:C (145). In response to OIR, increased levels of TLR2 and VEGF were observed in wild-type mice, whilst in mice deficient in TLR2, the levels of TGF-b, b-FGF and IL-6 were significantly reduced (139).

TLR4 levels are raised in response to retinal IR injury (13, 140, 142, 146–149) and hypoxia (7, 150). In particular, TLR4 levels are highly expressed in the INL (13, 146) and GCL (13, 142, 146) following I/R injury. TLR4 deficiency is associated with increased cell survival in the GCL and thickness of the INL following I/R injury (146, 151). I/R injury induces increased levels of tyrosine-protein kinase, Syk (SYK) and phosphorylated-Syk (146), in mice retina. This effect is lost in TLR4 KO mice treated with an SYK inhibitor, piceatannol, reducing I/R induced retinal damage (146).

TLR4 deficient mice develop reduced neovascularization (142) with preservation of normal vessel architecture in response to retinal I/R injury. This is associated with the increased secretion of TLR4-dependent inflammatory factors such as VEGF, NF-kB, b-FGF, TGF- β1, IL6 and IL-1β (142).

NLRP1 and NLRP3 play an essential role in the damage associated with retinal I/R injury. They are released in response to ischaemia and induce the release of IL-1β and IL18 (13). Ischaemia-induced NLRP3 is reduced by the administration of a TLR4 monoclonal antibody (clone HTA125) (13) and puerarin (149), which partially halts the development of retinal I/R injury.

Treatment of *in vitro* human retinal microvascular endothelial cells (HRMEC) with a TLR4 antagonist, TAK-242, reduces MAPK4-mediated inflammatory responses (148). Administration of TAK-242 in an OIR model has been shown to reduce the area of OIR avascularity, attenuate neovascularization, and increases vascular density (148). Furthermore, TAK-242 and anti-VEGF treatment have been shown to improve the vascular density and reduce aberrant angiogenesis in OIR mice (148).

I/R injury is characterised by the thickening of retinal layers due to oedema and disordered cell arrangement. Inhibition of TLR4 using N-acetyl serotonin (NAS) or TAK-242 (140) reduces the retinal thickening and pro-inflammatory cytokines, such as IL-1β in I/R (140).

Both CRVO and BRVO are associated with the elevated systemic levels of pro-inflammatory molecule, heparinase (152). Serum heparinase level and activity have been correlated with serum TLR4 and TLR2 levels (152).

MD2, a TLR4 co-adaptor molecule, plays a crucial role in the response to retinal I/R injury by forming an NADPH oxidase 4 (NOX4)-MD2-TLR4 complex. I/R stimulated release of HMGB1 activates downstream pathways which increases the intracellular ROS levels leading to cellular apoptosis (153). Inhibition of MD2 by L2H17 (154) reduces I/R injury by reducing apoptosis and suppression of oxidative stress-induced RPE cell death (154). In addition, MD2 inhibition reduces inflammatory injury during I/R through suppression of TBHP induced activation of the TLR4 pathways (154).

**Figure 5:**
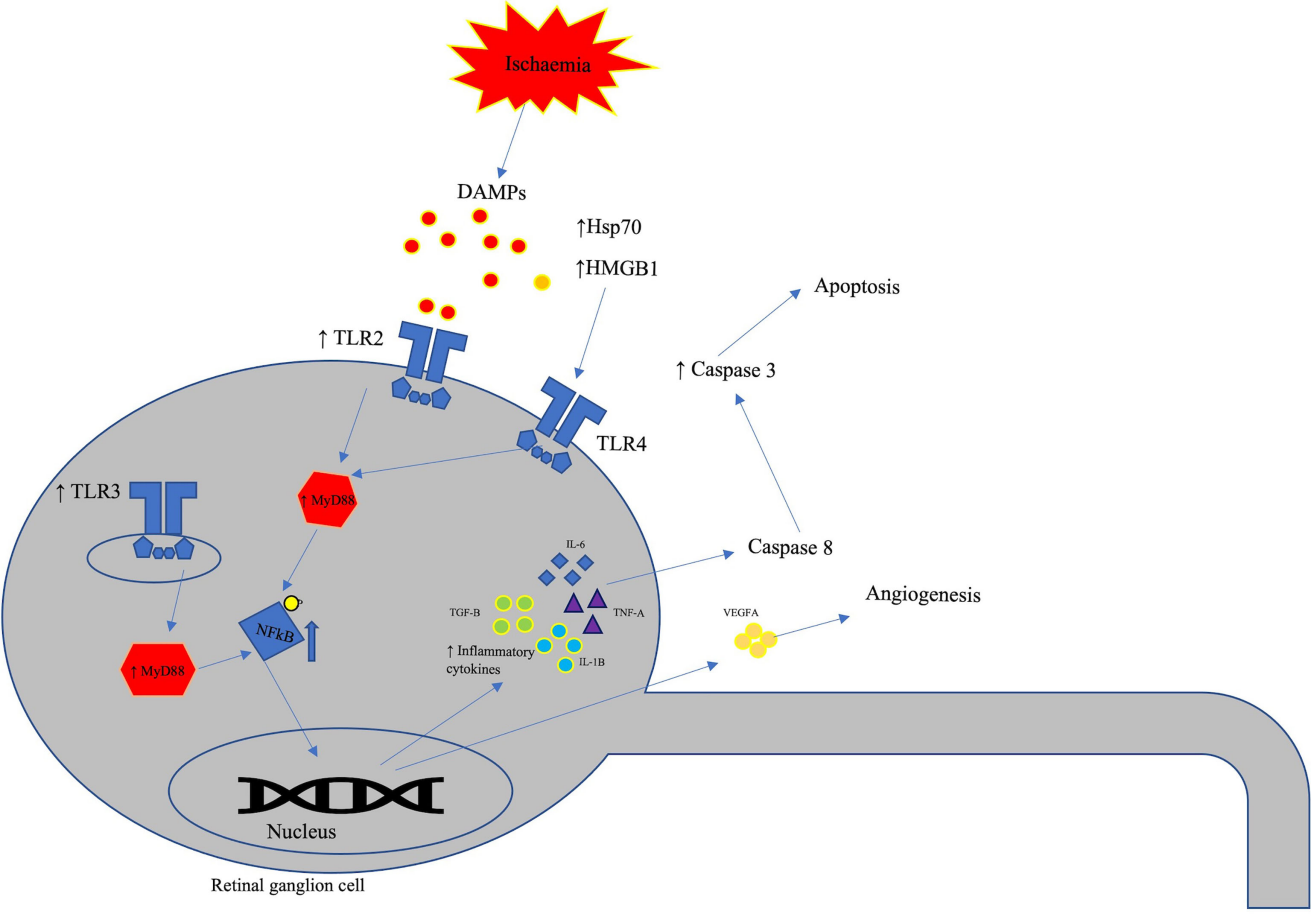
Schematic representation of TLRs involvement in retinal ischaemia. In response to retinal ischaemia, various damage associated molecular patterns (DAMPs) such as HSP70 and HMGB1 induces inflammatory response *via* the TLR4-MyD88 pathway. HMGB1 binds to TLR4 receptors leading to retinal ganglion cell loss and activation of inflammatory pathways. Retinal ischaemia also elevates the levels of TLR-1 to -3 in retinal ganglion cells leading to pro-inflammatory tissue damage.

Retinal ischaemia triggers the release of various DAMPs such as HSP70 and HMGB1, these are known to trigger cellular apoptosis *via* TLR mediated pathways (**Figure 5**). Retinal ischaemia has also been shown to induce increased levels of TLRs and there is a substantial amount of evidence showing an association between TLR4 levels and retinal ischemia. Retinal neovascularization following ischaemic injury is also influenced by TLR mediated pathways.

### 2.4 Retinal Dystrophies

#### 2.4.1 Retinitis Pigmentosa

Retinitis pigmentosa (RP) represents a group of retinal dystrophies with genetic inheritance. They are a frequent cause of hereditary blindness, affecting approximately 2 million people worldwide (155). RP consists of a wide range of heterogeneous conditions resulting from >3000 mutations affecting more than 60 individual genes (155). The different RP mutations have similar pathological characteristics, primarily a physiological dysfunction of photoreceptors, leading to their death. The continued loss of photoreceptor cells leads to continued stimulation of microglial proliferation, sterile inflammation, and further disease progression. Unfortunately, there is no identified treatment for RP, although gene therapy has been proposed as a potential management option.

As TLRs are a crucial component of the innate immune response and initiation of inflammatory pathways, their relevance in the sterile inflammatory pathways of late RP is important.

##### 2.4.1.1 TLRs in Photoreceptor Survival

In an RP model based on Pde6b (rd10) and Rho^P23H/+^ (P23H/+) background showed increased production of TLR2/MyD88 mediated IL-1β compared to wild type mice (155). Furthermore, RP mice with heterozygous TLR2 mutation were unable to improve photoreceptor survival. While those with TLR2 deficiency showed improved visual function and photoreceptor survival (155).

In a mouse model of inherited retinal degeneration, inhibition of MyD88 has been shown to reduce the photoreceptor apoptosis and functional loss (156). MyD88 inhibition also reduces microglia/macrophage infiltration in the neurosensory retina (156). Retinol dehydrogenase 8 (RDH8) and ATP-binding cassette transporter 4 (ABCA4) deficient mice that develop cone-rod dystrophy (CORD) showed increased expression of TLR signalling elements (157). ABCA4 and RDH8 deficiency have been associated with the development of features of CORD and worsened by light exposure. Additionally, TLR3 deficient mice failed to exhibit the signs of retinal damage (157).

### 2.5 Uveitis

#### 2.5.1 Background Information

Inflammation of the uvea, known as uveitis, can be triggered by infectious or non-infectious mechanisms. Uveitis can manifest in various conditions such as Behcet disease, ankylosing spondylitis, Vogt-Koyanagi-Harada, amongst others.

A short overview of TLR associations of uveitis is included in this review because of the common association between posterior uveitis and retinal inflammation which is broadly manifested as chorioretinitis. Our initial search criteria did not include uveitis and TLRs in general *per se*, but we investigated the relationship between the conditions that include posterior uveitis as part of ‘chorioretinitis’.

#### 2.5.2 TLRs in Uveitis

##### 2.5.2.1 Inflammatory Pathways

Patients with retinal vasculitis secondary to Behcet disease and idiopathic uveitis showed elevated levels of type 1 IFN (IFN A/B) than healthy individuals (158). Furthermore, levels of adhesion molecules such as soluble intracellular adhesion molecule-1 (sICAM-1) and soluble E-selectin were higher in patients with retinal vasculitis compared to healthy individuals (158). An *in vitro* investigation showed that retinal endothelial cells produced sE-selectin, sICAM-1 and IFN-β in response to TLR3 activation by poly I:C (158), implying that TLR3 activation may play a role in raised inflammatory markers in Behcet disease and idiopathic uveitis.

##### 2.5.2.2 Inherited Genetic Risk

A study was performed on the relationship between copy-number variants in the TLRs and various uveitis-related conditions, which included 400 patients with Vogt-Koyanagi-Harada syndrome, 400 Bechet disease patients, 400 patients with acute anterior uveitis and 600 healthy patients (159). The frequency of copy number variations in TLR1-3, TLR5-7 and TLR9-10 were compared between the patient groups, where only TLR7 was shown to have variation (159). One copy of TLR7 had a significantly increased frequency in male patients (p = 0.021) with Behcet disease, and two copies in female patients (p = 0.048). This result was confirmed in a second study with 587 Behcet’s disease patients compared to 1000 healthy control subjects (159) as a single copy in males (p = 1.14 x 10^-6^) and females (p = 9.12 x 10^-5^) (159). No association was found between TLR7 copy number variants and VKH or AAU.

Another study reported by (160) included 400 patients with Behcet’s, 400 patients with VKH, 400 patients with acute anterior uveitis, 400 patients with paediatric uveitis and 600 healthy controls, investigated the relationship between TLR2, TLR4, TLR8 and TLR9 (160). Behcet disease was associated with a higher frequency of the *TLR2-rs2289318* A and C allele (p=0.048, p = 0.008) and *TLR2-rs3804099* CT genotype (p = 0.005). This association was confirmed in a second state study consisting of 438 Behcet disease patients and 1000 healthy subjects; *TLR2-rs2289318* A and C allele (p=0.001, p = 6.89E-06) and *TLR2-rs3804099* CT genotype (p = 2.426E-06).

The relationship between TLR9 polymorphisms rs352140, rs352139, and rs187084 and VKH was investigated in a population of 94 patients and 125 healthy controls (161) and reported no statistically significant association.

The existing literature suggests that TLR polymorphisms do not appear to play a role in VKH or acute anterior uveitis. However, TLR2-rs3804099 and TLR2-rs2289318 polymorphisms appear to contribute to the risk of developing Behcet disease in a cohort of Han Chinese patients. Furthermore, copy number variants in TLR7 also appear to be associated with an increased risk of Behcet’s disease, with at least one contributing to risk in males and two contributing to risk in females.

### 2.6 Potential Ligands

#### 2.6.1 Tetramethylpyrazine

Chuanxiong, otherwise known as *Ligusticum wallichii* Franchat, is a Chinese herbal medicine containing the bioactive component 2,3,5,6 tetramethylpyrazine (TMP). It is used in traditional Chinese medicine to treat various ocular diseases such as glaucoma, DR, and AMD, acting through an unknown mechanism.

TMP pre-treatment has been shown to inhibit CD68 upregulation induced by LPS (162). TMP has been shown to inhibit endotoxin (lipopolysaccharide)-induced retinal inflammation (TNF-α, IL-6 and IL-1β) in rat retinal microglial cells *via* inhibition of the TLR4/NF-kB pathway (162).

#### 2.6.2 Puerarin

Puerarin, an isoflavone glycoside, is the bioactive component of the traditional Chinese herb known as Pueraria. Puerarin has been shown to have a protective role in DR and is thought to do so by attenuation of the NLRP3 inflammasome stimulation through inhibition of AB1-40 (149). In I/R injury, the administration of puerarin reduced the degeneration of rat RGC and attenuated the oxidative stress *in vivo*. This was thought to be mediated through the suppression of the TLR4/NLRP3 crosstalk mechanisms (149).

#### 2.6.3 Progranulin

Progranulin is a growth factor that regulates inflammatory responses, axonal growth and nerve cell survival (150). Cobalt chloride-induced retinal hypoxia in mice retina is associated with raised levels of HIF-α, VEGF, TLR4, and NOX4 (150). Ischaemia induced inflammation was attenuated by progranulin administration through a reduction of leukocyte adhesion and down-regulation of the TLR4-NOX4 signalling pathway (150).

#### 2.6.4 Apocynin

Apocynin is a methoxy-substituted catechol derived from *Picrorhiza kurroa*, which primarily inhibits NADPH oxidase dependent oxidative stress (163). Apocynin reduces hyperglycaemia induced retinal apoptosis, restores retinal morphology, and reduces oxidative stress through what was presumed to be *via* TLR4-NF-kB signalling axis (163).

#### 2.6.5 Gastrodin

Gastrodin, also known as 4-Hydroxybenzyl alcohol 4-O-beta-D-glucopyranoside, is the active component of the Chinese herb *Gastrodia elata* Blume (81), which has been previously shown to have anti-apoptotic and anti-inflammatory effects. Gastrodin inhibits hyperglycaemia induced HREC apoptosis by regulation of SIRT-1 induced inhibition of TLR4 signalling mechanisms (81).

#### 2.6.6 Paeoniflorin

Paeoniflorin is a monoterpene glucoside derived from the root of *Paeonia lactiflora* plant. It has been shown to exhibit immunomodulatory effects on microglial cells (84). High glucose has been shown to induce MMP-9 *via* HMGB1/TLR4 signalling in BV2 microglial cells (84). Hyperglycaemia induced MMP-9 is inhibited by the administration of paeoniflorin *via* induction of SOCS3 (negative regulator of TLR signalling cascade), leading to inhibition of TLR4 signalling (84).

#### 2.6.7 Wogonin

Wogonin, also known as 7−dihydroxy−8−methoxyflavone, is the active ingredient isolated from the roots of the *Scutellaria baicalensis* Georgi plant, commonly referred to as Huang-Qin (164). Administration of Wogonin to ARPE-19 cells has been shown to reduce LPS/TLR4 induced inflammatory mediators such as IL-1β, IL-8, IL-6, COX-2, iNOS and TNF-α (164).

## 3 Conclusions

Over the past two decades, there has been a great deal of progress in the understanding of TLRs in general, and their role in the eye, especially retinal diseases.

TLRs contribute to inflammatory pathways that occur in ischaemic retinal diseases, DR, and AMD. There appears to be a relationship between genetic polymorphisms and the risk of developing DR and AMD. In particular, the *TLR3- Asp299Gly* with DR, and *TLR4-rs4986790/TLR3-rs3775291* with AMD associations have been documented. However, the full extent of these relationships remains unclear and require further research that includes larger and different population groups. There is only limited research on TLR associations in inherited retinal degenerations, although inhibition of the TLR/MyD88 axis has been shown to improve the cell survival. Furthermore, it is important to consider that cell-lines and in particular non-human cell lines have limitations when extrapolated to other situations.

Most retinal diseases with described TLR association are non-infectious. It is, therefore, likely that the DAMP release from tissue damage due to drusen and associated molecular changes in AMD, or hyperglycaemic and associate conditions need to be closely evaluated for further clues.

Several studies have suggested that different substances may potentially reduce inflammation/apoptosis mediated *via* the TLRs. A greater understanding of TLR mechanisms in retinal diseases will allow us to identify potential new molecular targets for the treatment and diagnosis of these conditions. Identification of TLR modifiers or biomarkers (e.g., HMGB1 or DAMPs) in the vitreous and/or retinal tissue of patients with retinal disease will provide essential cues to develop selective or targeted inhibitors against them. Combining anti-VEGF therapies with TLR inhibition may provide a longer-lasting treatment in retinal vascular disease (such as neovascular AMD and DR) compared to anti-VEGF blockage alone.

## Author Contributions

All authors have made significant contributions to the manuscript. Conception and design: WA and IM; acquisition, analysis and interpretation of data: OT-L, IM, and WA; drafting and finalisation of manuscript: OT-L, IM, WA; final approval:OT-L, IM, WA.

## Author Disclaimer

The views expressed in this article are of the author and do not necessarily reflect the position or policy of the institutions they represent.

## Conflict of Interest

The authors declare that the research was conducted in the absence of any commercial or financial relationships that could be construed as a potential conflict of interest.

## Publisher’s Note

All claims expressed in this article are solely those of the authors and do not necessarily represent those of their affiliated organizations, or those of the publisher, the editors and the reviewers. Any product that may be evaluated in this article, or claim that may be made by its manufacturer, is not guaranteed or endorsed by the publisher.
